# Perceptions of black and minoritised ethnic occupational therapists on mentoring: A survey

**DOI:** 10.1177/03080226231209817

**Published:** 2023-11-15

**Authors:** Anita Atwal, Vimal Sriram, Elizabeth Anne McKay

**Affiliations:** 1 School of Allied and Community Health, London South Banking University, London, UK; 2 Director of Allied Health Professionals, University Hospitals Bristol and Weston NHS Foundation Trust, Bristol, UK; 3 Deputy Theme Lead, Collaborative Learning and Capacity Building Theme, NIHR ARC Northwest, London, UK; 4 Head of Occupational Therapy, School of Nursing, Midwifery and Social Car, Edinburgh Napier University, Edinburgh, UK

**Keywords:** Mentorship, BME, occupational therapy, ally

## Abstract

**Introduction:**

Mentorship is perceived as a mechanism to enhance career progression. Within occupational therapy, there is little research to demonstrate the effectiveness of mentoring on career success and no research has explored its relevance for black and minoritised ethnic (BME) occupational therapists. This research explored the experience of mentoring for career progression from a BME perspective using a survey.

**Methods:**

An online survey was conducted with occupational therapists in the United Kingdom who identified as BME. The primary recruitment method was a convenience sample via a BME network and through its other links. Content analysis and descriptive statistics were used to analyse and report the data.

**Findings:**

In all, 54 BME occupational therapists completed the survey. Most BME therapists had never requested a BME mentor, but most wanted a BME mentor. Active allyship was viewed as an important part of mentorship when mentored by a white therapist.

**Conclusion:**

This research is the first study in occupational therapy to examine the mentoring experiences and needs of BME therapists. It is a call for action to recognise and reorient the approach and understanding of the structures and experiences of BME mentorship.

## Introduction

In occupational therapy and other allied health professions, there has been an absence of research to explore the impact of ethnicity on career advancement (Atwal et al., 2021, 2023). One mechanism to facilitate career advancement is mentorship. Mentorship is thought to have two distinct functions (Kram, 1983):

1. Includes sponsorship, promoting exposure and visibility, coaching, protecting and
2. A psychosocial/personal development aspect such as role modelling, friendship and counselling.

For this study, we have used the definition of *‘A “mentor” describes a more senior person who takes an interest in the sponsorship of a more junior person and often have expertise and experience and knowledge and want to see mentees achieve career success’* (Scandura, 1998).

Mentoring systems are in place within health and social care settings as it is perceived to have a positive effect on the retention and career advancement of mentees (Rotondo and Perrewé, 2000). Within the UK, this is of importance since there is clear evidence that occupational therapists and other allied health professionals from minoritised backgrounds are not securing leadership positions (NHS England and NHS Improvement, 2022). There is a growing awareness of racism that occupational therapists are subjected to as part of everyday professional practice (Atwal et al., 2021; Beagan et al., 2022). Racial bias is also evident from service users’ perspectives; a recent scoping review explored racial bias from the perspectives of patients from minority backgrounds and healthcare providers, the review found that there were misconceived perceptions of low intelligence, patients from minority ethnic backgrounds were often not given sufficient information, staff were perceived to be more polite to white patients and staff from minority backgrounds were subjected to racism in terms of verbal and non-verbal communication (Hamed et al., 2022).

As part of addressing inequality within occupational therapy, it is important to determine whether structures in place allow all occupational therapists to progress in their careers. What we do not know is whether the processes and structures are inclusive to meet the career goals and expectations of black and minoritised ethnic (BME) occupational therapists. For this study, we have chosen to use the term BME occupational therapists as defined by the Law Society (The Law Society, 2022) to include persons from black, South Asian and East Asian ethnicities.

Minoritised ethnic (or the similar term ‘racially minoritised’) has been recommended more recently as it recognises that individuals have been minoritised through social processes of power and domination rather than just existing in distinct statistical minorities. It also better reflects the fact that ethnic groups that are minorities in the UK are majorities in the global population.

In the UK, allied health professionals (AHP) can be one of 14 distinct professional groups including occupational therapists (Health and Care Professions Council, 2023). A recent scoping review (Atwal et al., 2023) identified two studies that included BME AHP, examining mentorship using different models (Browne et al., 2013; Koberg et al., 1998). Koberg et al (1998) describe the traditional one-to-one mentoring model but give no specific data to refer to which AHP were from a minority background, and that white participants (*N* = 288) outnumbered Hispanic (*N* = 26) and African-American (*N* = 19) participants. Browne et al. (2013) examined peer mentorship with Aboriginal and non-aboriginal health professionals. What these studies suggest is that we may want to pay more attention to cross-race mentoring. Koberg et al. (1998) suggest inequalities in access to mentoring, with white professionals accessing mentoring more in comparison to Hispanic or African-American professionals. Both studies refer to the importance of psychosocial aspects of mentorship. In addition, Browne et al. (2013) suggest that cross-race mentoring could be a mechanism to increase confidence in working together and improve awareness of culture and race issues for AHP.

Ethnicity could be an important variable within mentoring: since it requires trusting, close didactic exchanges between mentors and mentees (Richard et al., 2017). Relational demography considers the extent to which employees are similar or dissimilar on factors such as ethnicity and gender (Guillaume et al., 2017). It is embedded in the similarity-attraction paradigm (Lankau et al., 2005) which suggests that persons are attracted to others, who they perceive as like them, which, in turn, can enhance the mentoring relationship. Similarly, social identity theory suggests that characteristics such as ethnicity, gender and class are used to develop relationships and a sense of connectivity (Hogg and Terry, 2000). Mentees who perceive themselves as being like their mentors report more positive outcomes from their relationship than those who do not have these perceptions (Eby et al., 2013). Findings from a study by Godshalk and Sosik (2003) included 217 working professionals from different professions, they found that the greater the similarity between the mentor and mentee, the greater the likelihood for the provision of psychosocial support, career development and role modelling. If mentees and mentors are mismatched in relation to personality, values and working styles this can cause mentoring relationships to be ineffective (Eby et al., 2000). Age has also been found to be an important characteristic of mentoring relationships. Older employees, within a university, experienced less career mentoring, had shorter relationships and were closer in age to their mentor, and reported more mutual learning than younger persons experiencing mentorship. Younger persons reported experiencing more mentorship from younger persons as their age increased (Finkelstein et al., 2003). It is, however, still unclear what the perceptions of BME occupational therapists’ current experiences are of mentoring for career progression.

This study explored mentoring from BME occupational therapists’ perspectives to identify what mentoring structures exist and if current mentoring structures are inclusive and credible for BME occupational therapists. This will ascertain what is currently available and whether changes are needed to ensure equity in the mentoring process to facilitate career progression and personal growth.

## Method

This study is part of a larger investigation on mentoring and safe spaces for UK-based BME occupational therapists. A steering group including occupational therapists from different sectors (health, education and social care) was established to advise on research processes and procedures. It should be noted that two out of the three members of the research team were BME occupational therapists with one researcher identifying as white. Ethical approval for the study was granted from a London University. This research study followed the CHERRIES Checklist for reporting on online surveys (Eysenbach, 2004).

A cross-sectional survey was conducted between May and July 2022. An online survey was developed based on a scoping review of the literature (Atwal et al., 2023) and the findings from four co-production workshops with BME occupational therapists. A summary of the themes found from the scoping review and co-production workshops is as follows:

1. Disparities in relation to the type and amount of mentoring
2. Disparities in relation to demographics on the mentoring process
3. Differences in relation to the aim of mentoring and its relevance
4. Uncertainty about the outcomes of mentoring on career progression

These themes were used to construct an online survey with closed- and open-ended questions, including demographic background, employment and safe spaces and mentorship. All questions, including demographic questions, did not require completion if the participant did not wish to do so. The survey’s usability (readability, language and relevance), face validity and technical functionality (in an online format) were evaluated by members of the steering group prior to it being launched. The estimated time to complete the questionnaire was less than 30 minutes.

We used an online survey site (https://www.onlinesurveys.ac.uk/about/) (formerly Bristol Online Survey [BOS]) that used an electronic link and QR code to access the participant information sheet and consent form. The primary recruitment method was a convenience sample via BAME OT UK (https://www.bameot.uk/), an affinity group of BME occupational therapists from the UK and through its networks. The BAME OT UK group promotes diversity and inclusivity, challenges the lack of equity and promotes anti-racism both within the profession and society and its members meet monthly. Social media such as X (formerly Twitter) was used to increase the reach of BME occupational therapists as not all BME occupational therapists are members of BAME OT UK. A Twitter chat was hosted to talk about mentorship to occupational therapists and we used other social media channels via key influencers and BME networks. In addition, an advertisement was placed in this research study funder’s newsletter. The time span for taking part in the survey was initially open for a month but the survey links stayed open for up to 2 months to allow for participation following advertisement and social media campaigns.

Participants were included if they were occupational therapists working in academic, independent, health or social care sectors in the United Kingdom and identified as BME. To capture the experience of mentoring, we encouraged participation from both mentors and mentees.

### Analysis

The closed questions were analysed using descriptive statistics. The open-ended questions were analysed using content analysis. Content analysis was used to examine the words used by BME therapists and decide whether the words used had a common relationship. In short, to try and identify relationships amongst concepts in the responses since individual concepts by themselves do not have intrinsic meaning (Morse and Field, 1995). The method by Erlingsson and Brysiewicz (2017) was followed to avoid bias. All the surveys were read to get a feel of the responses, initially looking at the frequency of the words and phrases, to understand the different words used and the meaning attached. Two researchers independently coded half of the survey text. Multiple meetings were held to ensure that the coding process was consistent and how sentences that had potential covert messages were interpreted. We used content analysis for concept development (model building) (Hsieh and Shannon, 2005) based on participant responses.

## Results

In total, 54 occupational therapists who identify as BME, from diverse backgrounds completed the survey. Table 1 outlines the ethnicity of respondents with most therapists identifying as Black African (*N* = 27, 50%). In all, 51 therapists identified as female, and 15 respondents were aged between 20 and 29, 22 respondents were aged between 30 and 39, 7 were between 40 and 49, 9 were aged between 50 and 59, and 1 respondent was over 60 years of age. Most of the respondents were working full time (*N* = 42, 78%) at the time of the survey.

**Table 1.** Ethnicity of respondents.

Ethnic category	Number of participants
Black Caribbean	6
Black African	27
Other Black	1
Mixed Race	6
Asian Indian	5
Asian Chinese	5
Other Asian	2
Yoruba	1
South American	1

The participants worked in diverse settings and with different providers. Most therapists worked in an National Health Service (NHS) acute hospital (*N* = 33, 61%) or in an NHS mental health setting (*N* = 14, 26%) with others in higher education, social care and independent sector settings. Respondents’ highest level of education was degree level (*N* = 24, 44%), closely followed by master’s level (*N* = 20, 37 %). A small number had a diploma (*N* = 2, 4%), a postgraduate diploma (*N* = 6, 11%) or a doctoral degree (*N* = 2, 4%). There was a range in relation to work experience, with 15 therapists (28%) being newly qualified, 8 (15%) reported being a Band 6 (grade achieved normally after 1–2 years of work), 15 (28%) reported being at a Band 7 (specialist post), 6 therapists (11%) reported being at a Band 8 post (specific research and leadership) with a further 3 (6%) therapists being in senior positions. Three respondents (6%) were employed within universities in teaching roles and one was employed in a school. Three of the respondents did not indicate their current work or role experience.

## Experience of mentorship

Distinct types of mentoring were available to BME occupational therapists. The majority of therapists had peer mentoring (*N* = 23), reverse mentoring (*N* = 10) and group mentoring (*N* = 3). Most participants accessed mentoring themselves (*N* = 16) and others were offered mentorship opportunities by their managers (*N* = 8). One therapist perceived that they were not given equal access to mentoring because of their gender and ‘*colour of my skin’*. Our survey found that slightly over 50% (*N* = 28) of BME mentors reported supporting someone in their career development.

Other options for mentoring were also suggested: two therapists proposing that there should be an advisory board of members rather than a single mentor. Some therapists perceived it would be beneficial to meet prior to mentoring for rapport building, protected time within work and flexible mentoring such as walking and talking. The two factors cited most frequently were that mentoring should be confidential (*N* = 14) and that it should be non-judgemental (*N* = 14). Negative experiences were reported and included mentoring that has no depth or shallowness (*N* = 3) as well as time issues (*N* = 1).

Participants valued their personal safety and empathised with the need for mentoring to allow therapists to discuss issues without fear of retribution and ‘*no one telling you they did it so you can too’.* Therapists did value honesty without judgement:

I know the advice is in my best interest, even if I don’t necessarily like it with another saying ‘Being able to express views from my unique perspective as BME without fear of repercussions. Often feel I must bite my tongue and self-censor as a minority.

Therapists highlighted the importance of collaborative allyship.

Mentor who is knowledgeable about systematic racism and bias and proactively engages with these issues with me.

Some BME therapists perceived mentoring could be virtual (*N* = 2), challenging (*N* = 4) and emphasised the need for privacy (*N* = 1), the need to know when mentors are available (*N* = 1), able to leave mentoring at any time (*N* = 1), trust (*N* = 1) and signpost to careers as well as providing tips and support.

### Rationale for a BME mentor

In relation to whether it was important to have a BME mentor, the majority perceived it was extremely important (*N* = 21), very important (*N* = 18) or somewhat important (*N* = 11). Most BME therapists had not asked their employer for a BME mentor (*N* = 48), with some therapists (*N* = 27) not being provided with a BME mentor. Some respondents had looked for but were unable to find a BME occupational therapist mentor (*N* = 23). One BME therapist stated.

Being lovely is not enough. I would like someone who is critically inclined. Deeply aware of socio-political issues, particularly regarding BME communities. Such criticality is seldom found in OT, from my experiences. The focus tends to be on how wonderful and naturally client centred OTs are.

One therapist believed a BME mentor would open opportunities for them, others believed it would enable them to have shared understanding and offer non-judgemental support (*N* = 3). One therapist wrote ‘No *need to hold back feelings or being judged on how I felt as a minority in the workplace’*.

Three participants identified the importance of dealing with specific aspects related to racism such as microaggressions and discrimination. Another therapist emphasised the need to see someone from a BME background that has experienced career progression and someone they can *‘relate to*’.

The importance of ‘*connectivity*’ was perceived as a key factor in building relationships (*N* = 3). One person perceived that the idea of shared experience means that ‘this *can enable better understanding of being a BME OT’.* A BME mentor would enable BME therapists to talk about aspects of racism such as microaggression and find neutral support (*N* = 1), whilst another perceived that a BME mentor and mentee could talk about successes and failures. One therapist wrote.

I would appreciate someone who is familiar with daily BME experiences and the systemic barriers that are faced, both internally and externally. The internal aspect is very important, because it is often the identity, we have accepted about ourselves, that can be detrimental to success and confidence. This is difficult to explain to someone who is not intimately acquainted with such lived experiences.

Similarly, another respondent wrote.

My identity, experiences and talents tend to be quite overlooked in comparison to my white colleagues. And my white colleagues don’t face the barriers to moving up the career ladder that I do. Therefore, I need a BME mentor. Only they can begin to understand and coach me to climb the ladder.

Additionally, 18 participants reported that they mentored someone who identified as BME.

## Safe spaces

Safe spaces are protected places where brave and sometimes difficult conversations can occur. In all, 17 therapists reported they did not have a safe space at work. One therapist responded that a safe space needed to be separate from management structures and another that it should be outside of the work environment. A participant referred to the importance of a *‘safe space’* to discuss experiences and to *‘navigate white spaces’*.

A safe space was where difficult conversations took place but a place where BME therapists talked about concerns with ‘confidence’, it was a place which could manage ‘strong feelings’ and a place to discuss inequalities but ‘don’t tell you to deal with inequality’.

## Understanding and validating experience

Regarding being able to speak to issues that matter to them, BME occupational therapists reported that during mentoring they were able to discuss issues that mattered to them such as racism in the workplace (*N* = 10), microaggression (*N* = 12), allyship (*N* = 7), cultural issues (*N* = 16) and checking and validating experiences (*N* = 15). Participants highlighted the importance of having representation and support from someone who has had similar experiences (*N* = 6) and an understanding of cultural differences (*N* = 2) which could enable more awareness of the challenges faced by BME occupational therapists.

Participants perceived that a BME mentor may have a better understanding of who they are. One wrote ‘You *need to experience being black. . . walk in my shoes to know where I can [and] want to go’.* Another stated, ‘*It is important for the mentor to have awareness and insight into the difference a BME OT experiences’.* The word identity was used by therapists (*N* = 2) as well as shared understanding and shared perspectives of issues that BME persons encounter in the workplace (*N* = 6).

One therapist *highlighted* the importance of discussing issues of ‘*discrimination and microaggressions with someone who looks like me, without judgement’*. The issue of validation was reported by some therapists (*N* = 4).

## Access to a white mentor

Some BME therapists (*N* = 6) reported that it was not so important that they should have access to a black mentor. These therapists perceived that it was the level of knowledge that mattered as opposed to ethnicity. A participant emphasised the importance of personality. One person perceived that differences exist between different BME persons:

Just because someone is BME does not mean that I will automatically get along, as there are so many shades of ‘BME’.

Participants perceived that it was important to have a choice. There was a view that white voices would allow different issues to be explored from different perspectives. One therapist wrote, ‘*It should be on both directions. If you are a mentee, you should be a mentor to someone else. The chain should spread across the board’*.

Some participants perceived that a white mentor would not understand their cultural experience. One therapist stated.

I just feel as a Black person you want to be able to receive validation with regards to a vast majority of things including culture, the way you were brought up. Black people generally have had an experience that they can and will share with you as another Black person. As a Black person in a white man’s world things seem to be geared to suit whiteness, western centric, individualism and lacking historical understanding. For years we have had a type of mentoring that clearly has not been conducive because we [are] still fighting for EQUITY.

There was a view from participants, that it was important for white mentors to acknowledge the differences between BME therapists *‘Don’t assume we are all the same or have the same experiences because both [sic] BME’*. There was a view that a mentor needed to help with well-being and boundary setting ‘*someone who can help me learn my boundaries in terms of self-care whilst also speaking out’*. By contrast, another therapist was of the opinion.

As much as I like racism being called out and discrimination issues raised, I do not wish to have a mentor who brings it up all the time. I would prefer our mentoring time to be protected and the discussion around social issues being more openly treated.

## Personal characteristics of mentors

Participants also described the characteristics needed to be a BME mentor including:

- Similar role and experiences and interest in the mentee,
- Senior in the field, knowledgeable and experienced with the system,
- Vision and leadership skills,
- Enthusiasm to share expertise,
- Managed to succeed despite obstacles,
- Optimistic, trustworthy, respectful, compassionate,
- Good listener,
- Empathetic, reflective and non-judgemental.

For some, an ideal mentor was a therapist who was an expert (*N* = 3) and knowledgeable (*N* = 2).

For career progression, BME occupational therapists wanted a mentor who would promote sponsorship, provide access to social networks/networking and connections to external opportunities. As well as, providing constructive feedback to support career progression, offer support to navigate systems, demonstrate fairness and equity. One therapist wrote,

To be themselves, know they are black (non-white) strong powerful, well read, experienced and knowledgeable and can and will disrupt things in the face of opposition even from people who claim to be on their side.

## Mentoring outcomes

Most occupational therapists who have had access to mentoring perceived that mentoring met their expectations (*N* = 25), with nine participants perceiving that it did not. Therapists reported mentoring was a positive outcome (*N* = 36). Therapists perceived that mentoring did enable them to achieve their professional goals (*N* = 21), impacted networking opportunities (*N* = 15) and highlighted visibility within their organisation (*N* = 23). BME occupational therapists did report that in the mentoring experience, they were able to discuss leadership (*N* = 19), as well as new perspectives (*N* = 15). One participant also perceived that mentoring highlighted their failures.

Over half of the participants reported that they were able to discuss career progression (*N* = 28). In the qualitative comments, additionally, BME occupational therapists commented that mentoring focused on career development (*N* = 5). Although BME therapists reported that they were able to speak about issues that mattered to them, nine therapists reported this was not part of their mentoring experience. Suggestions for ways to improve mentoring included: formalising the relationship (*N* = 4), increasing the frequency of mentoring (*N* = 3), more availability (*N* = 2), objectivity (*N* = 1), commitment (*N* = 1), structure and planning (*N* = 1) and regular meetings (*N* = 1).

## Gender and mentoring

The participants did not perceive that having a mentor from the same gender was important with only six reporting that it was extremely important and or very important. By contrast, the majority (*N* = 50) said it was not so important, or somewhat important or not at all important. In the qualitative comments, some BME occupational therapists perceived that having another person of the same gender was important if you wanted to gain knowledge about advancing your career whilst pregnant and or your career as a mother (*N* = 4) and or similar challenges and experiences (*N* = 8). In addition, trauma issues could mean that it was difficult to talk to a male professional. A number of BME occupational therapists emphasised that the experiences of BME men and women were different (*N* = 4) and that ‘*Males and females are not singing from the same sheet at times, regardless of colour’*. Most perceived that the BME element was more important than gender.

## Discussion

This is the first study in occupational therapy, and within allied health professions, which has explored mentoring from the perspective of BME occupational therapists. Mentorship is important for workforce retention since mentees who report experiences of lower-quality mentoring report higher intentions to leave their jobs, compared to those who have a more positive experience of mentoring (Ragins et al., 2000). This survey found that the majority of BME occupational therapists were generally positive about the impact of current mentoring programmes on career and personal outcomes. This survey found that for most BME therapists, it was important to have a BME mentor, although therapists did not request this when participating in mentorship programmes. BME occupational therapists were also mentoring persons from BME backgrounds. Having a BME mentor for a BME mentee in occupational therapy may have a better outcome, similar to two meta-analyses that found mentees who perceived themselves to be similar to their mentors, reported a positive mentoring outcome (Eby et al., 2013; Ghosh, 2014). However, some therapists perceived that knowledge was more important than race or gender and highlighted that differences exist within individual ethnic groups in the wider BME community. Nonetheless, access to a BME mentor was difficult and it is important to build capacity, if BME therapists need access to BME mentors that they perceive will meet their career progression goals.

It is clear that there is a need to consider cross-race mentoring strategies as there is a limited number of BME therapists. For the mentor/mentee relationship to develop, both individuals needed to support the preferred strategy. Thomas (1990) examined 22 mentor and mentee relationships (African American and White). He found that strategies used for managing ethnic differences were either denying or suppressing such discussions; however, discussing it openly and whether both parties preferred the same strategy influenced the kind of relationship that developed.

This survey’s findings identified that psychosocial support is viewed as important; nonetheless, some BME occupational therapists did not have a safe space within the work environment. Importance was given to shared values and personal connections within the mentoring relationship (Straus et al., 2013). A key skill for a mentor is the willingness to share personal and professional experiences and to act as advocates for mentees (Burgess et al., 2018). Furthermore, the findings suggest that mentors need to be active allies. It has been suggested that active allies may have a better understanding of their own privileges (Straus et al., 2013). There are four primary motivations to be viewed as an ally (Radke et al., 2020).

(a) Those persons who are authentic and want to make a difference.
(b) Allies who will support a disadvantaged group if their own status is maintained.
(c) Personal motivations in which persons want to be seen as doing the right thing.
(d) Morality motivation where one’s personal moral compass is a key motivating factor.

This study found that to make mentorship meaningful it needs to be authentic, confidential, understanding, and offer non-judgemental support. The findings indicate that empathy was viewed as the most important skill for both an ideal mentor and a BME mentor. Evidence from the literature supports this view, with increased self-disclosure increasing rapport for both mentees and mentors in interracial mentoring outcomes (Leitner et al., 2018). Sponsorship is an important aspect of both mentorship and allyship, sponsorship means that a mentor is able to provide opportunities for personal and career development and promote their mentee’s skills and abilities (Sharma et al., 2019). Participants in our study perceived that a mentor should be well connected to promote networking opportunities. It has been suggested that mentoring should be seen as a process supporting formal networking (primary), supported by secondary mentoring for example indirect mentorship for additional support (Termini et al., 2021).

### Strengths and limitations

This survey is a first of its kind investigating mentoring perspectives among BME occupational therapists in the UK. The survey questions were informed by a scoping review, focus group and steering group discussions to capture what was important, what was known and what needed further investigation within this research area.

This study was conducted through an online survey and despite various communication channels being used, did not draw many respondents. This could have been because the survey timing was during the recovery phase of the COVID-19 pandemic. None of the participants were employed within research, third sector and or private healthcare provider settings and engaging these populations would be beneficial for future work.

## Conclusion

This research has highlighted that most mentored therapists had their expectations met. However, there is a need for further research to explore cross-race relationships within mentoring and the need to ascertain whether mentoring does have a positive effect on career outcomes. Whilst BME therapists may want access to a BME mentor, consideration needs to be given to why BME occupational therapists have not requested one. Furthermore, how can a critical mass of therapists from diverse backgrounds be built to meet this need? The importance of safe spaces was identified and the need for allyship to be authentic.

The findings from this study can be used to form guidance for mentoring for both cross-race mentoring and mentoring generally. There is a need to provide guidance and support to ensure white therapists are committed to the principles of allyship and formulate a model so that equity is assured for all within occupational therapy and the broader allied health professions. Further explorations of the effectiveness of different models of mentorship and creating safe spaces for conversations for BME occupational therapists are needed.

**Key findings**

- Mentoring is important to occupational therapists.
- There is a lack of BME mentors to support those who wish to have a BME mentor.
- Availability of safe spaces in the workplace is important for BME occupational therapists.

**What the study has added**

This is the first study to investigate UK BME occupational therapists’ perspectives on mentoring for career progression. It highlights the range of mentoring approaches and that there is an unmet need for BME occupational therapists to have BME mentors.
